# Detection of mesenchymal stem cells senescence by prelamin A accumulation at the nuclear level

**DOI:** 10.1186/s40064-016-3091-7

**Published:** 2016-08-26

**Authors:** Chiara Bellotti, Cristina Capanni, Giovanna Lattanzi, Davide Donati, Enrico Lucarelli, Serena Duchi

**Affiliations:** 1 Osteoarticular Regeneration Laboratory, 3rd Orthopaedic and Traumatologic Clinic Prevalently Oncologic, Rizzoli Orthopaedic Institute, via di Barbiano 1/10, Bologna, 40036 Italy; 2 Department of Biomedical and Neuromotor Sciences, University of Bologna, Bologna, Italy; 3 Institute of Molecular Genetics - Unit of Bologna, CNR-National Research Council of Italy, Bologna, Italy; 4 Laboratory of Musculoskeletal Cell Biology, Rizzoli Orthopaedic Institute, Bologna, Italy

**Keywords:** Mesenchymal stem cells, Lamin A, Prelamin A, Senescence, Cell- and tissue-based therapy

## Abstract

**Background:**

Human mesenchymal stem cells (MSC), during in vitro expansion, undergo a progressive loss of proliferative potential that leads to the senescent state, associated with a reduction of their “medicinal” properties. This may hampers their efficacy in the treatment of injured tissues. Quality controls on MSC-based cell therapy products should include an assessment of the senescent state. However, a reliable and specific marker is still missing. From studies on lamin-associated disorders, has emerged the correlation between defective lamin A maturation and cellular senescence.

**Findings:**

Primary cultured hMSC lines (n = 3), were analyzed by immunostaining at different life-span stages for the accumulation of prelamin A, along with other markers of cellular senescence. During culture, cells at the last stage of their life span displayed evident signs of senescence consistent with the positivity of SA-β-gal staining. We also observed a significant increase of prelamin A positive cells. Furthermore, we verified that the cells marked by prelamin A were also positive for p21^Waf1^ while negative for Ki67.

**Conclusions:**

Overall data support that the detection of prelamin A identifies senescent MSC, providing an easy and reliable tool to be use alone or in combination with known senescence markers to screen MSC before their use in clinical applications.

**Electronic supplementary material:**

The online version of this article (doi:10.1186/s40064-016-3091-7) contains supplementary material, which is available to authorized users.

## Background

Human mesenchymal stem cells (MSC) have raised high hopes in various therapeutic applications and their use is currently tested in about 500 clinical trials (www.clinicaltrials.gov). For many clinical approaches these cells are usually expanded in vitro prior to their utilization. However, MSC can undergo only a limited number of cell divisions under standard culture conditions, and it has been demonstrated that during in vitro proliferation they suffer a progressive and continuous process of aging (Wagner et al. 2008) that affects the proliferation and multilineage differentiation potential (Banfi et al. 2000; Kim et al. 2010), the immunomodulatory properties (Sepúlveda et al. 2014), the secretory profile (Coppe et al. 2010), the gene expression profile, and the epigenetic signature (Schellenberg et al. 2011; Yoo et al. 2013). These changes may impact the efficacy of MSC to treat injured tissues. For this reason, it is generally recommended in clinical applications to use MSC that have been cultured only for a restricted amount of time, fixing a threshold that limits their number of passages or population doublings (de Girolamo et al. 2013; Wuchter et al. 2014).

Cellular senescence is a complex process, which manifests with different, multifaceted phenotypes depending on the species, the cell type, and the senescence-inducing stimulus (Kosar et al. 2011). This lack of a unique signature implies the necessity for a mindful selection of the proper marker or panel of markers when assessing the senescence status of in vitro cultured MSC. The detection of SA-β-gal activity is the most common assay for the evaluation of cell senescence due to the availability of well-established protocols and commercial kits, and the abundant bibliography reporting its use. However the limitations of this marker are universally recognized (Crowe et al. 2014; Debacq-Chainiaux et al. 2008; Wagner et al. 2009). Other markers of senescence, opportunely reviewed by de Jesus (de Jesus and Blasco 2012), such as telomere shortening, Senescence-Associated Heterochromatin Foci (SAHF), Promyelocytic Leukemia Protein Nuclear Bodies, and Senescence-associated secretory phenotype (SASP) have been proposed over years. Recently, new approaches specifically focused on MSC have been suggested. Shibata et al. explored the expression of the p16^INK4A^ gene, suggesting its methylation state could be monitored as a surveillance against the transformation of MSC during culture (Shibata et al. 2007). Koch et al. proposed instead the use of the DNA-methylation changes observed at specific CpG sites in MSC and fibroblast to track the state of cellular senescence (Koch et al. 2012).

We moved from the observation of the phenomena that influence or lead to aging in humans, in order to identify a reliable and specific marker of the senescence phenotype of MSC.

In this regard, alteration of the nuclear lamina is known as one of the cellular changes observed in physiological aging (Vlcek and Foisner 2007; Lattanzi et al. 2014). Of particular interest is the *LMNA* gene that encodes two components of the nuclear envelope: lamin A and C. The maturation of lamin A is an elaborate process which involves several consecutive steps including: farnesylation, the proteolytic cleavage of three N-terminal amino acids, the carboxymethylation and the final removal of additional fifteen N-terminal amino acids including the farnesyl group. The final step is exclusively catalyzed by the zinc-metallopeptidase ZMPSTE24 encoded by the *FACE*-*1* gene. Mutations affecting different steps or actors of the maturation process, which elicits the accumulation of wild-type or mutated prelamin A, are associated with progeroid laminopathies or lipodystrophy (Broers and Ramaekers 2006; Davies et al. 2011). These diseases, including the Hutchinson-Gilford progeria syndrome that is characterized by premature aging, mainly affect tissues of mesenchymal origin, suggesting a link between prelamin A and MSC senescence.

The existence of this correlation was supported by the work of Scaffidi and Misteli. Their results demonstrated that the accumulation of wild type or mutant lamin A by means of expression vectors or drugs leads to an accelerated aging of human fibroblast and immortalized MSC (Scaffidi and Misteli 2008). Our goal was to verify this correlation the other way around, and so where replicative senescence of primary MSC culture leads to prelamin A accumulation. The presence of lamin A precursors in cells after their prolonged in vitro culture or in tissue specimens from aged donors was already observed by other investigators, but their analysis was focused on Vascular Smooth Muscle Cells (Ragnauth et al. 2010).

As far as we know, a general and robust detection analysis of lamin A precursor in MSC that have naturally exited the replicative cycle in normal culture conditions has never been reported. Therefore, in our work we used primary cultures of human MSC isolated from the bone marrow of healthy donors to investigate the presence of unprocessed lamin A precursor during early and late stages of in vitro cultures, with the ultimate scope of proposing a proper marker to detect senescent MSC.

## Methods

Primary human MSC were obtained from 3 non-oncologic patients (aged 20, 26, 6) during routine orthopedic surgical procedures.

Cell isolation and expansion is described in the Additional file.

### Definition of early and late stages of in vitro MSC culture

MSC were maintained in culture until they reached their maximal life span as evidenced by growth arrest (i.e. the cells failed to become confluent within 4 weeks of culture).

The number of population doublings (PD) for each passage was calculated using the formula: log_2_(N_1_/N_0_), where N_0_ is the number of cells seeded and N_1_ is the number of cells harvested at the end of the passage. Cumulative population doublings (CPD) were calculated as the sum of PDs over passages.

CPD curves were normalized with GraphPad Prism 6 Software to set the maximum CPD value as the 100 % of the cell line life-span. Early and late life-span stages were then identified by graphical interception on the CPD curve tracing horizontal lines at y coordinates equal to 50 and 80 % (Stenderup et al. 2003). Experimental observations were performed on cell samples at passages comprised in the “early stage” (life-span <50 %) or “late stage” (life-span >80 %).

### Senescence associated β-galactosidase assay

SA-β-gal activity was detected with a senescent cell staining kit (Sigma Aldrich, St. Louis, MO, USA) according to the manufacturer’s instructions. Briefly, the 40 mg/ml stock solution of 5-bromo-4-chloro-3-indolyl β-d-galactopyranoside (X-gal) was prepared in the laboratory by dissolving the X-gal powder (Sigma Aldrich, USA) in *N,N*-dimethylformamide. Once prepared, it was stored at −20 °C and used within a month, to ensure the accuracy of the assay.

Cells were seeded in a 24-well plate and cultivated until 60 % confluence. Plates were washed with PBS, fixed for 5 min at room temperature, and incubated at 37 °C overnight in a dry incubator with freshly prepared 1 mg/ml X-gal buffered solution. Cell nuclei were counterstained with 5 µg/ml Hoechst 33342 (Life Technologies, Eugene, OR, USA). Microphotographs of a minimum of six random fields for each sample were taken using an epifluorescence microscope (Nikon Eclipse TE2000-U, Amsterdam, Netherland) equipped with a Nikon DS-Vi1-U3 CCD color digital camera. Brightfield and fluorescent images were merged with NIS-D software (Nikon, Amsterdam, Netherlands) to count total and β-gal positive cells.

### Prelamin A detection and immunostaining

MSC grown on coverslips were fixed in cold methanol at −20 °C for 7 min. Samples were incubated with PBS containing 4 % BSA to saturate non-specific binding and incubated overnight at 4 °C with anti-prelamin A antibody (Santa Cruz Sc-6214) diluted at 1:100. Coverslips were then washed several times in PBS and incubated 1 h at RT with donkey anti-goat secondary antibody (Santa Cruz Sc-3853) diluted 1:100. After washes with PBS, the nuclei were counterstained with 4,6-diamino-2-phenylindole (DAPI). The slides were mounted with an anti-fade reagent in glycerol and observed. To measure the percentage of prelamin A positive cells, microphotographs of a minimum of 6 random fields were taken for each sample and prelamin A positive cells were manually counted. Imaging was performed using a laser-scanning motorized confocal system (Nikon A1R, Nikon, Amsterdam, Netherlands) equipped with an Eclipse Ti-E inverted microscope and four laser lines (405, 488, 561, and 638 nm). A Plan Apo VC 60x/1.4NA Oil DIC N2 objective lens was used. Images were processed using NIS-Elements AR 4.10.01 software (Nikon, Amsterdam, Netherlands).

As a positive control, accumulation of prelamin A was obtained using 25 μM mevinolin (M2147, Sigma) in complete growth medium for 18 h. Mevinolin is an isoprenoid synthesis inhibitor that causes inhibition of hydroxymethyl-glutaryl-synthase implicated in the farnesyl pathway. This compound elicits an accumulation of non-farnesylated (unprocessed) prelamin A, as reported in several studies (Mattioli et al. 2008).

To observe prelamin A nuclear distribution, confocal imaging was performed using the 488 nm laser line, and laser power was adjusted to minimize photobleaching. A Plan Apo 100x/1.4NA Oil DIC H objective lens was used. Zoom and field-of-view dimensions were adjusted to give a resolution of 1024 × 1024. *Z*-slices were acquired every 0.175 μm for a total of 31 steps. NIS elements software permits 3D rendering of the *Z*-slices.

Two double immunostainings of prelamin A with p21 or Ki67 were performed. The rabbit polyclonal anti-Ki67 antibody (Santa Cruz Sc-15402) was diluted 1:100 and incubated overnight at 4 °C while the rabbit monoclonal anti-p21antibody [Pierce p21 Waf1/Cip1 Antibody (R.229.6)] was diluted 1:300 and incubated overnight at 4 °C. Immunofluorescence microscopy was performed using a Nikon E600 epifluorescence microscope and a Nikon oil-immersion objective [100× magnification, 1,3 NA (numerical aperture)]. Photographs were taken using a Nikon digital camera (DXm) and NIS-Element AR software.

## Results and discussion

In the production of MSC-based cell therapy products, safety and potency evaluations are mandatory, and among other aspects, the senescent state of culture should be assessed.

Though alternatives to the classical SA-β-gal staining have been proposed (Shibata et al. 2007; Righolt et al. 2011; Koch et al. 2012) we believe an optimal marker for the identification of senescent MSC is not yet available. A correlation between accumulation of prelamin A and cellular aging has been already postulated (Scaffidi and Misteli 2008; Yu and Kang 2013; Yu et al. 2013). However, the main findings of the previous studies were mainly obtained through an induced accumulation of prelamin A with expression vectors or drugs. Our goal was to demonstrate that the spontaneous accumulation of prelamin A occurs in human MSC under a condition of replicative senescence induced by the prolonged in vitro culture.

**Fig. 1:**
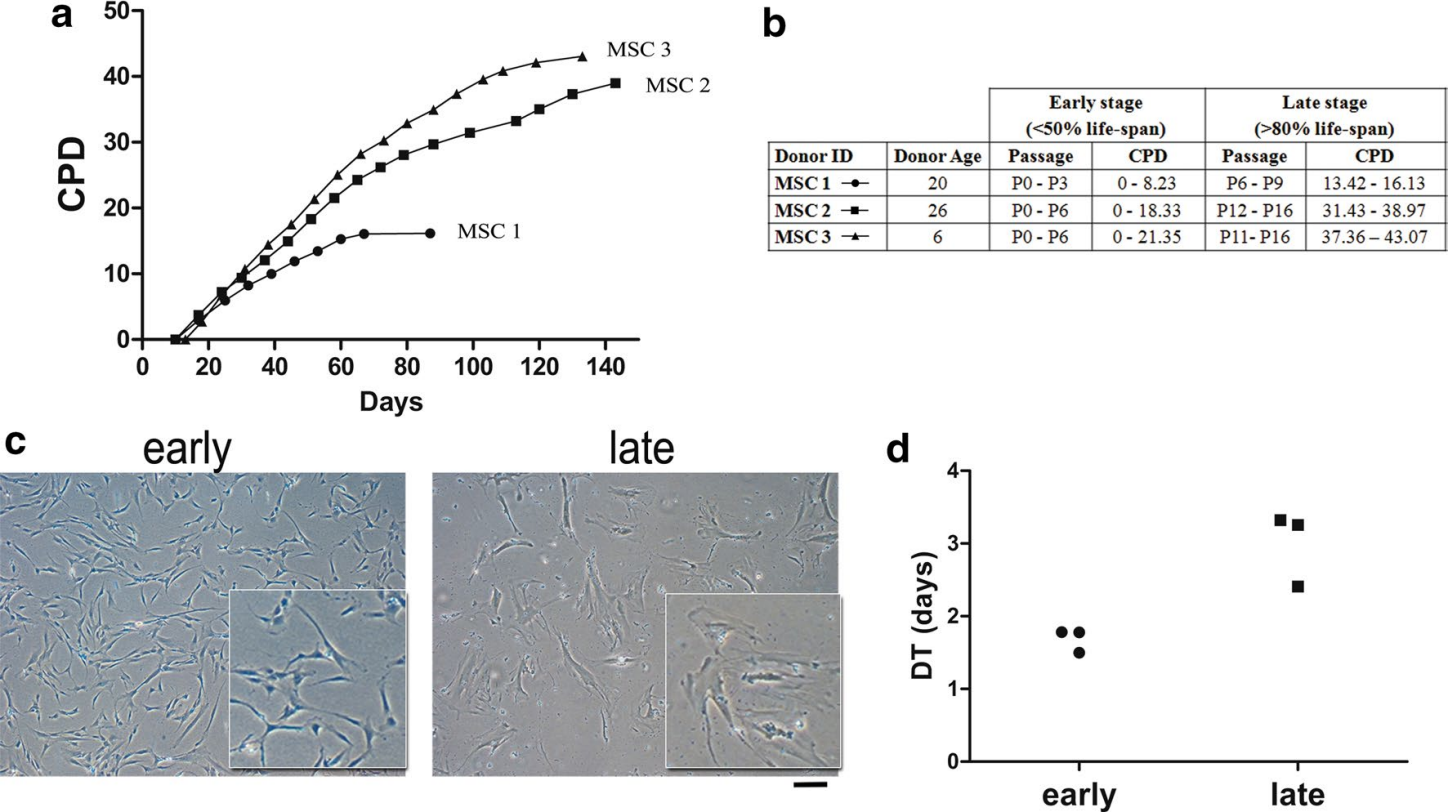
MSC undergo replicative senescence during in vitro expansion. **a** Cumulative population doublings (CPD) of cell cultures from three different donors. **b** Passages and PDT intervals corresponding to the early and late stages of each MSC cell line. The end of the late stage matches with replicative senescence of the culture. **c** Representative brightfield images of MSC at early and late stages. *White boxes* indicate areas of magnification highlighting the altered shape of senescent cells compared to the well defined spindle-shape of MSC at early stages. *Scale bar* 200 µm. **d** Doubling times (DT) calculated from proliferation assay confirm the remarked reduction of proliferation potential of cells according to the progression of life-span stages

MSC samples were isolated from the bone marrow of three healthy donors and maintained under standard culture condition until they reached replicative senescence in culture. As expected, when expanded in vitro each of them presented a distinctive growth curve and progression toward the Hayflick limit (Shay and Wright 2000) (Fig. 1a). Even if cells are isolated and cultured under the same conditions, the number of passages is evidently an unreliable indicator of cellular aging. Observations were therefore planned after the analysis of the growth curves of each cell line both at early and late stages (Fig. 1b), as described in the Materials and Methods section. The comparison of cells belonging to the same life-span stage attenuates the inter-donor variability and allows the observation of a coherent range of modifications such as a gradual change from a fibroblastic-like spindle shape to a large widespread morphology (Fig. 1c) and a reduced proliferation potential in the late stage (Fig. 1d). These morphological changes are associated with cytoskeleton alteration. In fact, β-tubulin and Vimentin immunostaining analyses (Additional file 1: Fig. S1A, B), performed at early and late stages demonstrated the altered polarization and disruption of cytoskeletal microtubules filaments of senescent cells (Geißler et al. 2012).

**Fig. 2:**
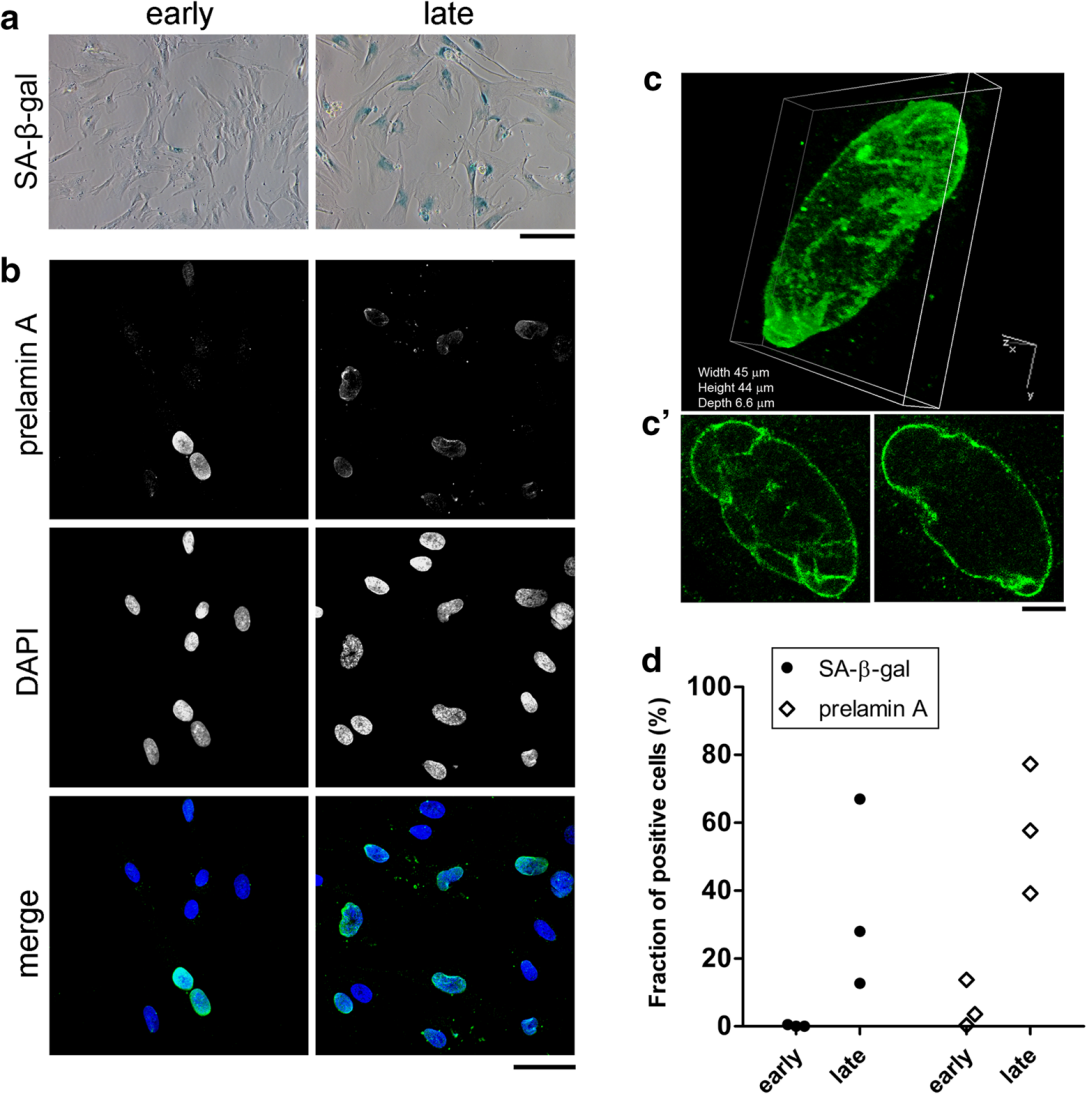
MSC under replicative senescence accumulate prelamin A. **a** Representative microphotograph of SA-β-gal assay performed on early and late stage cells. *Blue* staining indicates the presence of β-galactosidase activity in senescent cells. *Scale bar* 200 μm. **b** Cells at early and late stages were fixed and immunostained with a specific antibody against prelamin A and counterstained with DAPI. *Scale bar* 50 µm. **c** 3D digital rendering of Z-slices confocal images of a representative prelamin A positive cell at late stage. **c’** Single slice images of different Z-stacks illustrating the distribution of prelamin A, from surface (*left panel*) and from middle plane (*right panel*) point of views. *Scale bar* 10 µm. **d** Quantification of β-gal positive and prelamin A positive cells at early and late stages. *Blue* stained cells and Hoechst stained nuclei were counted in a minimum of six random fields to report the percentage β-gal positive cells at early and late stages. To count prelamin A positive and total cell numbers a minimum of six random fields was checked at early and late stages. Data are expressed as percentage of positive cells respect to the total cell count

The senescent status of the cell cultures at late stages was confirmed by the SA-β-gal assay that revealed an increased activity of the lysosomal β-D-galactosidase at the suboptimal pH of 6.0, compared to the cells at early stage (Fig. 2a). Similarly, the specific immunostaining for the full-length form of lamin A precursor (with an antibody directed to the C-terminal residue of the unprocessed protein) showed a higher number of cells positive for prelamin A at late stages (Fig. 2b). Confocal microscopy performed at high magnification confirmed a nucleoplasmic and rim localization of prelamin A together with an abnormal nuclear morphology (Fig. 2c, c’). From the single z-stacks representative of different planes along the nucleus (Fig. 2c’), multiple invaginations of the nuclear membrane are evident. Interestingly, the observation of an increased incidence of wrinkled nuclei in senescent cells was exploited by Righolt et al. to elaborate an imaging method that quantifies the intensity and curvature of the nuclear lamina to identify abnormal cells during aging, in vitro proliferation, and in lamina disorders (Righolt et al. 2011).

The manual count of the percentage of SA-β-gal or prelamin A positive cells over the total performed on the 3 MSC lines confirmed the qualitative results. The percentage of prelamin A-positive cells increases dramatically from early to late stages (from 6 to 59 %), with a trend similar to the one observed for the SA-β-gal staining (Fig. 2d). Though the trends were similar we can observe that the results from prelamin A staining indicate a slightly higher percentage of positive cells than for SA-β-gal assay both at early and late stage. The number of observations is insufficient to operate a statistical comparison, but the observed difference could indicate that the accumulation of prelamin A is a more sensitive marker to reveal senescent cells even at earlier stages. The detection of prelamin A positive cells at early stage is not entirely unexpected, as primary MSC culture are known to be composed by an heterogeneous population that might harbor among the active proliferating cells, resting, terminally differentiated or senescent cells (Sherley 2002; Whitfield et al. 2013).

In addition, the variability among MSC lines observed for SA-β-gal counting despite we performed our analyses at stages selected to reduce the inter-donor variability (Fig. 1), is reduced in the prelamin A scoring, making the latest a more steady marker.

The discordant results from the two markers might also be a consequence of the limitations and technical pitfalls inherent to the SA-β-gal assay caused by the limited specificity and instability of β-gal substrate, as already outlined by other investigators (Yang and Hu 2005; Lee et al. 2006; Debacq-Chainiaux et al. 2009). In this regard, the assessment of prelamin A accumulation can be performed with an easy available positive control, in order to check the technical quality of the immunostaining. The positive control for prelamin A consists in cells treated with mevinolin, a drug known to inhibit farnesyl production and to cause accumulation of unprocessed prelamin A (Lattanzi et al. 2014) (Additional file 1: Fig. S2). As expected from previous studies (Caron et al. 2007), treatment with mevinolin did not induce SA-β-gal expression (Additional file 1: Fig. S2). This is in agreement with our data showing that cells from centenarian individuals spontaneously accumulate prelamin A and display a more efficient response to oxidative stress-induced DNA damage (Lattanzi et al. 2014). These data support the view that prelamin A accumulation does not induce senescence per se, but it is triggered in response to stress stimuli in the attempt to counteract geroconversion. In particular, we observed in human fibroblast that the oxidative and replicative stress affect prelamin A processing, through the reduction of ZMPSTE24 expression (Lattanzi et al. 2014) and it could be speculated that a similar mechanism is involved in prelamin A accumulation observed in senescent MSC.

Biochemical evaluation of prelamin A was also performed by Western blotting analysis. Prelamin A, in accordance with results obtained by prelamin A immunofluorescence staining, is detectable at the predicted molecular weight in both senescent and mevinolin-treated MSCs (Additional file 1: Fig. S3).

**Fig. 3:**
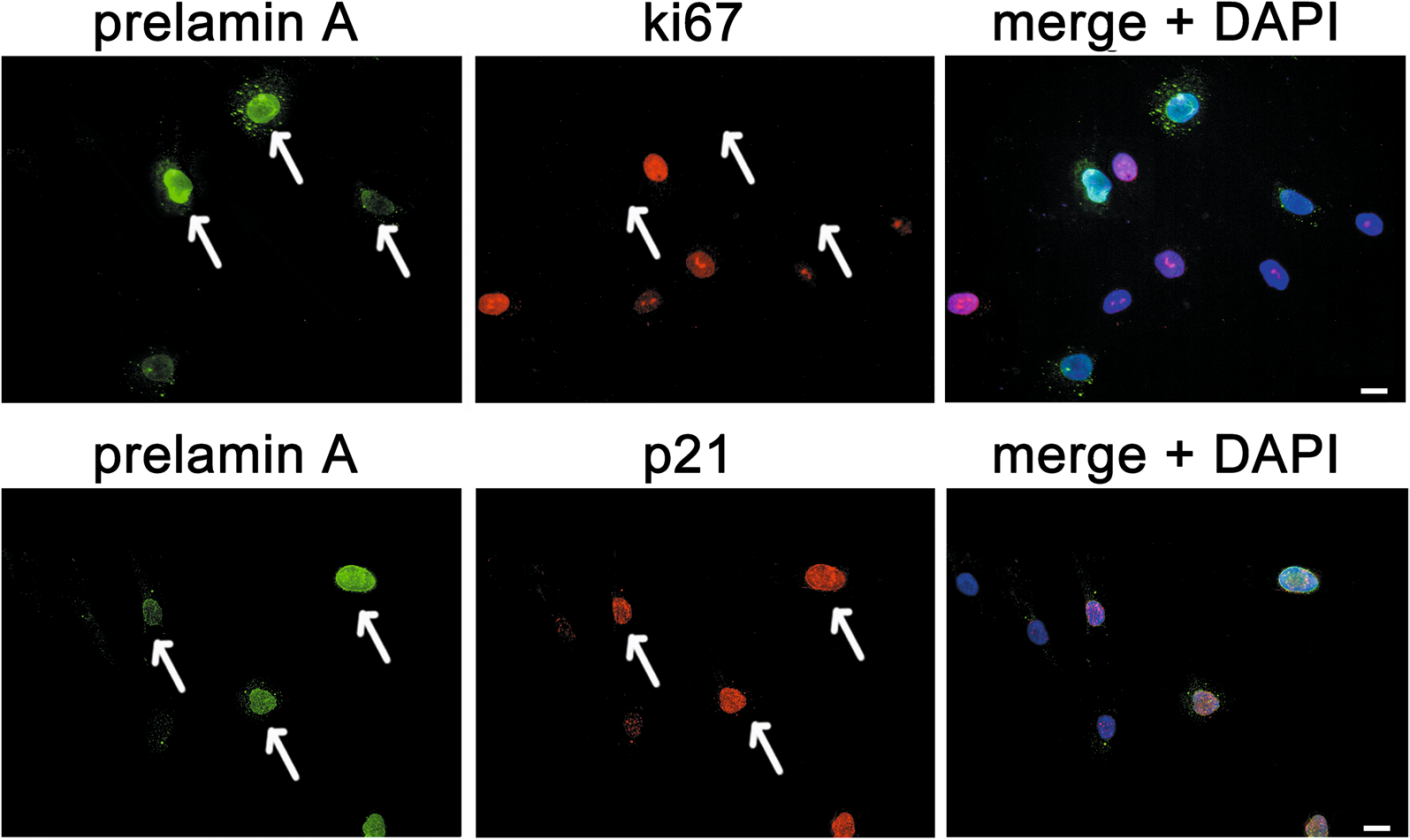
Cell-cycle arrested cells are positive for prelamin A. Representative images of MSC immunostained for prelamin A (*green*), Ki67, and p21 (both *red* in the *panels*). Cell nuclei were counterstained with DAPI (*blue*) and merged images are shown in the last column. Prelamin A positive cells (*arrows*) are negative for Ki67 and positive for p21. *Scale bar* 10 µm

We further checked the expression of p21^Waf1^ (*Cdkn1n* gene), a nuclear protein that indicates senescence-associated cell-cycle arrest (Kong et al. 2011), and Ki67, a marker of proliferating cells expressed in all active phases of the cell cycle (G_1_, S, G_2_). As hypothesized, cells positive for prelamin A showed p21^Waf1^ staining in nuclei and were negative for Ki67 (Fig. 3), confirming that prelamin A-labeled MSC underwent replicative senescence during in vitro culture.

Based on our observations, the nuclear accumulation of prelamin A identifies senescent cells in human MSC cultures.

It is worth noting that previous reports indicate that the accumulation of mutated forms or precursors of lamin A triggers a series of molecular changes that diminish the specific properties of the MSC such as the multilineage differentiation potential (Yu et al. 2013; Malashicheva et al. 2015) and the capacity to promote the repair of injured tissues (Infante et al. 2014). Therefore, prelamin A accumulation can be therefore considered a candidate marker for the detection of senescent cells during MSC expansion and can be exploited to discard aged cells, characterized by low differentiation and regeneration capacity, and thus prevent their release for clinical purposes.

## Conclusions

Despite the underlying molecular mechanisms are still unraveled, replicative senescence has evident consequences for cellular therapy. In this paper we have demonstrated that in human MSC cultured in vitro under standard growth condition, the detection of prelamin A identifies senescent MSC. Since the onset of senescence in a MSC culture is currently difficult to predict by the use of available markers, prelamin A staining could be successfully used to screen MSC populations before they are used for clinical applications alone or in combination with know and currently used senescence marker.

## Additional file

10.1186/s40064-016-3091-7 Supplementary data.

## Abbreviations

SA-β-gal: senescence associated β-galactosidase
MSC: mesenchymal stem cells
