# Supplementary material for: Detection of mesenchymal stem cells senescence by prelamin A accumulation at the nuclear level
Source: Springerplus. 2016 Aug 26;5(1):1427. doi: 10.1186/s40064-016-3091-7 (PMC5001959; doi:10.1186/s40064-016-3091-7)
Supplement: Supplementary file 1 — 10.1186/s40064-016-3091-7 Supplementary data. [file 40064_2016_3091_MOESM1_ESM.pdf]

Supplementary Materials and Methods

Isolation of MSC and long term expansion

MSC were isolated from bone marrow aspirates using gradient separation (Ficoll-Paque PREMIUM, density 1.073 g/mL; GE Healthcare, Uppsala, Sweden) and plastic adherence, as previously described (Pierini et al. 2013). After the gradient separation step, viable mononucleated cells were seeded (density: $4x10^5$ cells/cm^2) in complete medium composed of α-modified minimum essential medium (α-MEM; BioWhittaker, Lonza, Verviers, Belgium) supplemented with 20% lot-selected fetal bovine serum (FBS; Lonza, Basel, Switzerland), 1% GlutaMAX ™ (Gibco, Life Technologies, Paisley, UK), 1% Pen-Strep solution (Gibco, Life Technologies, Paisley, UK). The medium was changed after 48 h of culture, to remove non-adherent cells. When large confluent colonies covering more than 50% of the plate were observed, cells were detached by trypsinization (TripLe™ Select; Life Technologies) and cell number and viability were assessed with an automated cell counter (NucleoCounter®, ChemoMetec, Lillerød, Denmark). Isolated MSC were replated for expansion at a density of 2000 cells/cm^2 (passage 1) in the complete medium used for isolation w/o of the antibiotics. Medium was changed twice a week and cells were passaged when they reached 70% - 80% confluence.

Methylene blue proliferation assay (supplementary material)

Cell proliferation at early and late stages was measured by methylene blue assay. Briefly, MSC were seeded at 2000 cells/cm^2 in 96-well plate and incubated at 37°C in 5% CO_2 humidified atmosphere. After 1,3,6 and 10 days, cells were fixed by adding 10% formol-saline to each well for 30 min. Cells were then stained with filtered 1% (w/v) methylene blue in 0.01 M borate buffer (pH 8.5) for 30 min. Excess dye was removed and the wells were rinsed three times with 0.01 M borate buffer (pH 8.5). A volume of 100 µL of 1:1 mixture (v/v) of absolute ethanol and 0.1 M HCl was added to each well to elute the dye. The plates were carefully shaken and the absorbance at 650 nm (A650) was measured using a microplate reader (Synergy HT, BioTek Instruments Inc., Winooski, VT USA) and blank-corrected on control wells containing elution solvent alone. The number of cells at each time point was calculated by interpolation of absorbance values (from five replicates) against a standard curve. Then the proliferation curve of each cell line at early and late stage were fit in an exponential growth model by GraphPad Prism 6 software to obtain an estimation of the doubling times.

Cytoskeleton immunodetection

MSC grown onto glass coverslips were fixed for 7 min in ice cooled 100% Methanol. After three washes in PBS 1×, cells were incubated in blocking solution (PBS 1× + 5% BSA) for 30 minutes at RT. Primary antibodies diluted in blocking solution were added and incubated overnight at 4 °C. After three washes in PBS 1×, secondary antibody was added diluted in PBS 1× and incubated for 45 min at RT.

Primary antibodies used were: mouse anti-Vimentin (Sigma-Aldrich #V6630 [1:2000]) and mouse anti-βTubulin (Sigma-Aldrich #T4026, [1:50]). Secondary antibody used was anti-mouse IgG-Cy3 [Sigma-Aldrich #C2181,1:100] for both primary antibodies. Nuclei were stained by incubating cells with 5 µg/ml Hoechst 33342 (Life Technologies, [1:2000]) for 10 min at RT after secondary antibody step.

Coverslips were mounted after washes in PBS 1× in Fluoromount-G solution, and imaged using a laser-scanning motorized confocal system (Nikon A1R, Nikon, Amsterdam, Netherlands) equipped with an Eclipse Ti-E inverted microscope and four laser lines (405, 488, 561, and 638 nm). A Plan Apo VC 60x/1.4NA Oil DIC N2 objective lens was used. Images were processed using NIS-Elements AR 4.10.01 software (Nikon, Amsterdam, Netherlands).

Western blot analysis

Human MSCs were lysed in lysis buffer containing 20mM Tris–HCl, pH 7.5, 1% SDS, 1mM Na3VO4, 1mM PMSF, 5% β-mercaptoethanol and protease inhibitors. Proteins were subjected to SDS gradient gel (5-20%) electrophoresis and transferred to nitrocellulose membrane. Incubation with anti-prelamin A (Santa Cruz Biotechnology sc-6214) or anti-lamin A/C (Santa Cruz Biotechnology sc-6215) primary antibodies was performed overnight at 4°C. Incubation with anti-actin (Santa Cruz Biotechnology I-19) was performed for one hour at room temperature. Bands were revealed by the Amersham ECL detection system (Capanni et al. 2010).

Supplementary references

Capanni C, Cenni V, Haraguchi T, et al (2010) Lamin A precursor induces barrier-to-autointegration factor nuclear localization. Cell Cycle 9:2600–10. doi: 10.4161/cc.9.13.12080

Pierini M, Di Bella C, Dozza B, et al (2013) The posterior iliac crest outperforms the anterior iliac crest when obtaining mesenchymal stem cells from bone marrow. J Bone Joint Surg Am 95:1101–7. doi: 10.2106/JBJS.L.00429

Supplementary Figure 1

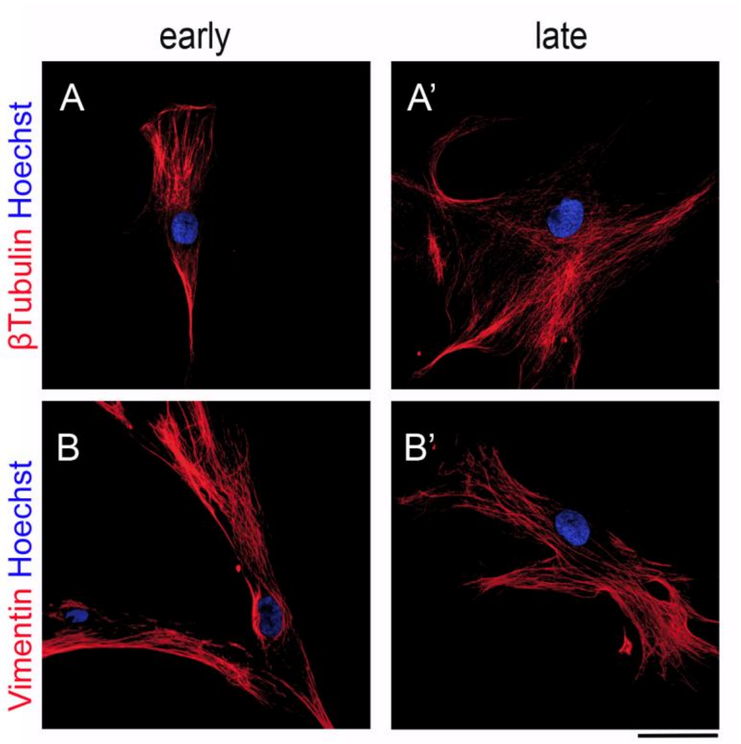

Suppl. Fig. 1. Alteration of morphology according to life-span stages. (A-B) Representative confocal images of MSC stained with β-tubulin (red in C) and Vimentin (red in D) and counterstained with Hoechst (blue in C and D) at early and late stage. Altered polarization and disruption of cytoskeletal microtubules is evident at late stage of in vitro culture. Scale bar=50µm.

Supplementary Figure 2

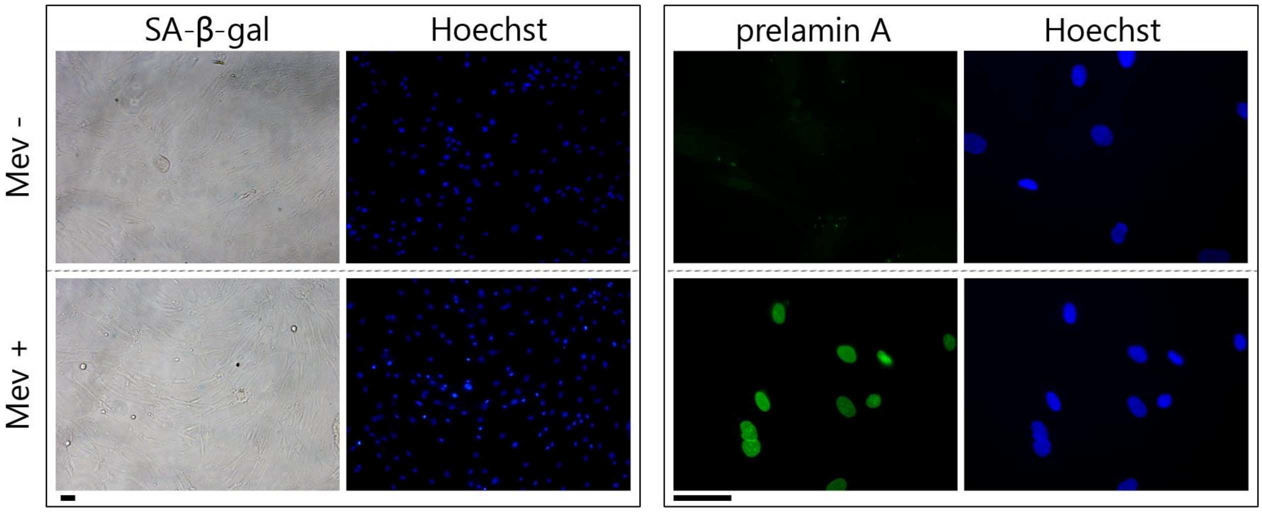

Suppl. Fig. 2. Mevinolin treatment do not induces SA-β-galactosidase activity. Representative microphotograph of SA-β-gal assay and prelamin A immunostaining performed on MSC treated for 18 h with 25 µM mevinolin in complete growth medium (Mev+) along with a control sample of untreated early stage MSC (Mev-). Mevinolin treated cells are strongly positive for prelamin A, while the detection of SA-β-gal activity shows a negative result identical to untreated cells. Scale bar = 50 µm.

Supplementary Figure 3

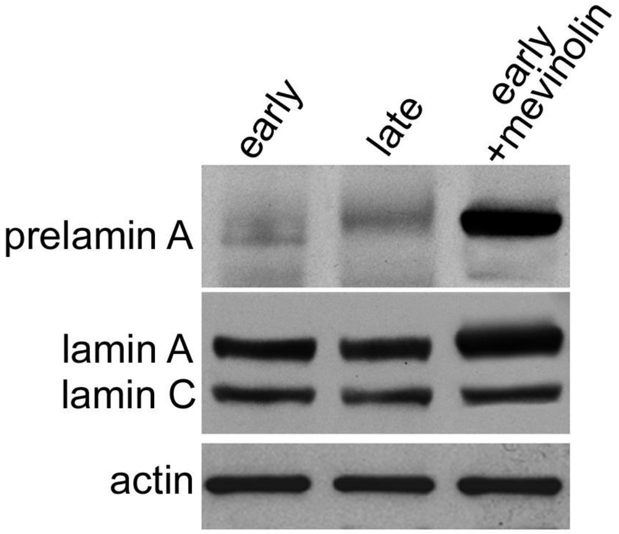

Suppl. Fig. 3. Western blot analysis of prelamin A accumulation in late stage MSC culture. Western blotting evaluation of prelamin A in early and late stage and MSC treated for 18 h with 25 μM mevinolin in complete growth medium. Total cell lysates were subjected to prelamin A (prelamin A) and lamin A/C (lamin A/C) detection. Actin was evaluated as protein loading control.
